# Imatinib for bleomycin induced pulmonary toxicity: a case report and evidence‐base review

**DOI:** 10.1002/ccr3.549

**Published:** 2016-04-01

**Authors:** Iouri Banakh, Alice Lam, Ravindranath Tiruvoipati, Ian Carney, John Botha

**Affiliations:** ^1^ Department of PharmacyFrankston HospitalPeninsula HealthFrankstonVic.Australia; ^2^ Department of Intensive Care MedicineFrankston HospitalFrankstonVic.3199Australia; ^3^ School of Public HealthFaculty of Medicine, Nursing and Health SciencesMonash UniversityClaytonVic.3800Australia

**Keywords:** Bleomycin, imatinib mesylate, intensive care, pulmonary toxicity, respiratory failure, treatment outcome

## Abstract

The evidence supporting therapy with imatinib for bleomycin‐induced pneumonitis (BIP) is equivocal. Further experience is needed to establish its role in BIP management. While it may be considered in the management of BIP, it is important to be mindful of the adverse effects including thrombocytopenia and gastrointestinal bleeding.

## Introduction

Bleomycin is an antibiotic chemotherapy agent with low myelosuppressive properties that is still frequently used for management of Hodgkin's lymphoma and testicular cancer 1, 2, 3, 4. Bleomycin clearance is dependent on bleomycin hydroxylase and the lack of this enzyme in lungs and skin predisposes patients to significant pulmonary toxicity 1, 2, 4. Pulmonary adverse events from bleomycin are some of the most common lung toxicities studied and frequently present as bronchiolitis obliterans with organizing pneumonia (BOOP), eosinophilic hypersensitivity, as well as interstitial pneumonitis with fibrosis 1, 2, 3, 4. Bleomycin interstitial pneumonitis (BIP) has been thought to be caused by direct endothelial damage of the pulmonary vasculature with severe inflammation and stimulation of the immune system, causing acute and chronic inflammation with progression to fibrosis 1, 4. Bleomycin pulmonary toxicity has been associated with significant mortality, particularly in patients receiving concomitant radiation therapy 1, 2, 3, 4. Many risk factors for development of BIP have been proposed and include oxygen therapy, high cumulative doses of bleomycin, renal impairment, smoking, mediastinal radiation therapy, patient age (>40 years), use of colony‐stimulating agents, and the administration method 1, 2, 3, 4. Several agents have been recommended for the management of BIP based on animal study results including interleukin (IL)‐1‐receptor antagonists, anti‐tumor necrosis factor (TNF)‐*α* antibodies, and inhibitors of transforming growth factor (TGF)‐*β* 1, 4. Corticosteroids at high doses are frequently used, but are thought to benefit BOOP and eosinophilic hypersensitivity rather than BIP, with the diagnosis of these conditions frequently confused 1. Arsenic trioxide has been trialed in management of BIP as it reduces TGF‐*β* and its downstream effects on differentiation of fibroblasts as well as type‐1 collagen deposition in pulmonary tissues 5. Similarly, imatinib suppresses the transformation of fibroblasts into myofibroblasts and reduces collagen deposition via inhibition of platelet‐derived growth factor and TGF‐*β* signaling 6. Arsenic studies have been confined to animal models, while imatinib has not improved outcomes in human studies of idiopathic pulmonary fibrosis (IPF), a condition that shares many biochemical and histological features with BIP 5, 6. A very small number of case studies have been published examining the use of imatinib in BIP and other forms of pulmonary fibrosis, with variable effects on outcome 7, 8, 9, 10, 11. These data were the rationale for using imatinib therapy in our patient.

## Case Study

The patient treated at our hospital was a 69‐year‐old male with comorbidities that included stage IV Hodgkin's lymphoma, hyperlipidemia, and coronary artery disease. His alcohol consumption was moderate and he was an ex‐smoker. He was diagnosed with Hodgkin's lymphoma 8 months prior to his hospitalization. His lymphoma chemotherapy consisted of cycles of doxorubicin, bleomycin, vinblastine, and dacarbazine (ABVD) over the 4 months preceding presentation with 28 day cycles and infusions on days 1 and 15 of each cycle. His prehospitalization results suggested a response to therapy with a positron emission tomography scan shortly before admission revealing no evidence of ongoing disease activity, but with bilateral pulmonary interstitial infiltrates on the scan. Chemotherapy treatment for the lymphoma caused significant weight loss, with his body mass index dropping to below 19 on admission.

**Figure 1:**
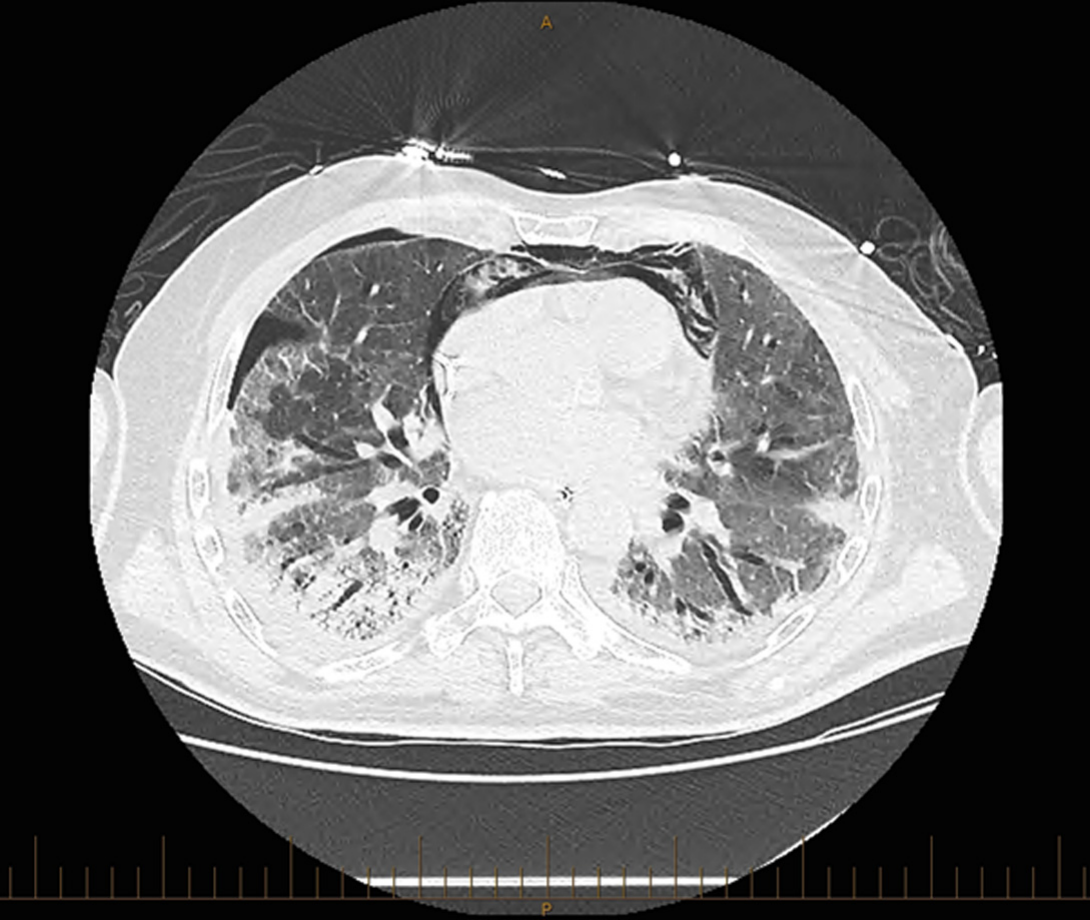
CT scan on day 3 showing a pneumomediastinum and a small right pneumothorax.

He initially presented to hospital, 5 days after his last dose of chemotherapy, afebrile with increasing dyspnea over a 2–3 week period, associated with a nonproductive cough, hypoxia, tachypnea, and pulmonary infiltrates on chest radiograph. His white blood cell count (28.7 × 10^9^) and C‐reactive protein (68 mg/L) were elevated. His renal, hepatic, and coagulation profile were within the normal ranges. Initial management included noninvasive ventilation, which he failed within 48 hours of admission requiring endotracheal intubation and invasive mechanical ventilation. Specimens were taken from blood, urine, sputum, and bronchial washings to exclude bacterial, fungal, and viral infections on different occasions during the admission. Ventilation was complicated by barotrauma as evidenced on a chest computer tomography (CT) scan performed on day 3 of hospitalization which revealed a large pneumomediastinum and a small right pneumothorax (see Fig. 1). Since the patient did not have any cardiovascular effects of pneumomediastinum or a significant deterioration in gas exchange, it was conservatively managed. The small pneumothorax was carefully monitored on serial chest X‐rays. Lung protective ventilation (assist control mode with a plateau pressure of 27 cm of water, tidal volumes of 300 mL [5 mL/kg tidal volume], and a respiratory rate of 24) was implemented to prevent worsening of pneumothorax. The pneumothorax completely resolved in 4 days with this treatment strategy. The chest CT scan documentation also indicated [sic] “extensive ground glass change throughout both lungs” when compared to a previously available scan results and showed [sic] “long‐standing mild to moderate compression fractures at T10 and T11” without destructive bone lesions. He was treated empirically with trimethoprim/sulfamethoxazole for Pneumocystis jiroveci pneumonia (PJP) prophylaxis and therapeutic azithromycin, piperacillin/tazobactam, and anidulafungin as the patient was immunocompromised due to completion of the last cycle of chemotherapy. Additional therapy included prednisolone 75 mg daily for pneumonitis, esomeprazole for stress ulcer prophylaxis, salbutamol for intermittent wheeze, enoxaparin for venous thromboembolism prophylaxis, and imatinib 100 mg three times a day for suspected BIP. After a poor response to the above therapies with worsening gas exchange and persistent hypercarbia, extracorporeal removal of carbon dioxide was considered but not commenced due to the presumed irreversible nature of lung injury and associated risks of bleeding secondary to anticoagulation. He was sedated with morphine and midazolam, and required low‐dose vasopressor support with a noradrenaline (norepinephrine) infusion.

After a few days of hospitalization, his serology for *Mycoplasma pneumonia* was negative and PJP prophylaxis was empirically escalated to a therapeutic dose given the lack of significant improvement with the antimicrobial and antifungal therapies while awaiting the microbiological results of bronchial washings. As there were concerns regarding the evidence of benefit and potential complications of imatinib, high‐dose corticosteroid therapy was recommended as an alternative to imatinib. Hydrocortisone was discontinued and replaced by methylprednisolone 1 gm daily IV for 3 days on day 8 of hospitalization while imatinib therapy continued. On day 9 of his ICU admission, he developed hematemesis and melena requiring blood transfusions and a proton pump inhibitor infusion (pantoprazole loading dose of 80 mg followed by 8 mg/hour infusion for 72 hours) was initiated for management of gastrointestinal hemorrhage. This occurred in the setting of therapy with methylprednisolone, imatinib, therapeutic doses of trimethoprim/sulfamethoxazole, and enoxaparin prophylaxis (40 mg once a day). The patient's platelet count also declined from 169 × 10^9^/L on the day of admission to 89 × 10^9^/L on the day of the hemorrhagic event. After the complication of gastrointestinal hemorrhage, imatinib and enoxaparin were ceased. Imatinib was a likely contributing factor with previously recognized adverse events of both thrombocytopenia and gastrointestinal hemorrhage 12.

Early on day 12, cisatracurium was commenced for worsening acute respiratory distress syndrome with increasing agitation and ventilator dyssynchrony (see Table 1) 13. The PJP polymerase chain reaction result from a bronchoalveolar lavage was negative, permitting cessation of trimethoprim/sulfamethoxazole treatment, while aciclovir was empirically added as a therapy for viral pneumonia given the ongoing deterioration. Following 3 weeks of treatment with the above mentioned therapies, the patient continued to deteriorate with worsening gas exchange and profound deconditioning of the patient. After discussion with oncologists and respiratory physicians, it appeared that the prospects of recovery were poor. The family of the patient at this stage were requesting for a comfort care given the prolonged hospital stay. A family meeting was held and palliation was advised and implemented. Four core biopsy samples were taken postmortem, three samples from the lungs, and one from the liver. The autopsy results showed expansion of the interstitium with collagen, scattered elastin fibers, and incorporated fibrin, suggesting a subacute process. In addition, there were occasional myofibroblastic foci scattered throughout the lung biopsies, suggesting slight temporal heterogeneity with no honeycomb formation. There was no evidence of fungal elements, no granulomatous inflammation, and no dysplasia or malignancy. The presentation was consistent with organizing diffuse alveolar damage, with minor temporal and spatial heterogeneity. In addition, there were changes within the pneumocytes suggestive of the cytological atypia described in bleomycin toxicity.

**Table 1:** Arterial blood gasses changes with cisatracurium^a^ use for acute respiratory distress syndrome

Day of admission	11	12 (before cisatracurium)	12 (after cisatracurium)	13
PaO_2_ (mmHg)	54	61	62	70
FiO_2_ (%)	45	50	50	40
PaO_2_:FiO_2_	120	122	124	175
pH	7.34	7.27	7.34	7.33
pCO_2_ (mmHg)	65	80	68	75
Base excess	6.4	5.6	7.6	8.9
SaO_2_ (%)	85.6	87.0	89.5	93.5

a Cisatracurium was initiated on day 12 of hospital admission due to worsening respiratory failure.

## Discussion

This case contributes to the limited literature describing the use of imatinib for treating BIP 7, 8, 9, 10. The current experience in using imatinib for BIP has had variable success. Three out five patients developed BIP after four cycles of bleomycin, while one patient presented after completing all six cycles of ABVD and one patient after a single dose of 90 mg of bleomycin in treatment of testicular cancer. Most patients had typical BIP presentation of dry cough, shortness of breath, and classic CT changes of diffuse alveolar damage and ground‐glass opacities similar to our patient 4, 7, 8, 9, 10. Unlike the four previously published cases, in our case imatinib therapy was initiated from the onset of hospitalization for presumed bleomycin pulmonary toxicity with concomitant broad‐spectrum antibiotic cover as the patient was recently treated with chemotherapy. The exclusion of infection is important under such circumstances as both high‐dose corticosteroids and imatinib cause further immunosuppression, placing patients at higher risk of pneumonia with PJP being an important differential diagnosis 4. As the patients with successful outcomes required 6 months of treatment with imatinib, the hemorrhagic and immunosuppressive effects of this agent should be carefully considered and monitored. Our case was complicated by thrombocytopenia with gastrointestinal bleeding. Imatinib is known to cause thrombocytopenia in more than 10% of patients with gastrointestinal hemorrhage being a known adverse event with an occurrence rate of 0.1–1% 12.

Bleomycin is used in animal studies for simulation of IPF and research into its treatment, and it has been suggested that treatments for IPF could be used for treatment of BIP due to a shared pathogenesis 14, 15. However, this has been disputed and suggested that only the early phases of both conditions share some similarities 15. There is some support for this view with many successful treatments in bleomycin animal models failing to show any benefit in IPF. The therapies that have not shown benefit in the management of IPF include corticosteroids, everolimus, warfarin, bosentan, interferon‐*γ*, etanercept, and imatinib 14. There must be caution in extrapolating any results from animal studies to humans 15.

Some case reports have suggested that high‐dose corticosteroids, including prednisolone at 1 mg/kg^4^, ^9^, ^16^ and methylprednisolone up to 1 gm/m^2^ for at least 5 days, with prolonged weaning may be successful in management of BIP 15, but only a few cases showed some benefit. Azathioprine has been used successfully as a corticosteroid sparing agent in a case of BIP, when preceded by high‐dose methylprednisolone 17. Extracorporeal membrane oxygenation (ECMO) therapy may be considered at an earlier stage in BIP‐suspected patients, with lower oxygen saturation targets, as oxygen therapy has been previously identified as a risk factor for the condition due to increased bleomycin toxicity through superoxide radical formation 1, 4, 10, 18. While there are no studies supporting the use of extracorporeal carbon dioxide removal specifically in bleomycin toxicity, it may be considered where ventilation is complicated by persistent of hypercarbia 19.

## Conclusion

This case report adds to the limited literature on the use of imatinib in BIP and highlights the potentially life‐threatening complications that may be associated with this agent. While the evidence supporting the use of imatinib for BIP is equivocal, judicious use of this agent may be considered in patients with BIP. Further experience on the use of imatinib is required to clarify the role of imatinib in BIP management.

## Conflict of Interest

None declared.
